# Unprecedented Haemorrhagic Stroke: A Rare Manifestation of Atypical Haemolytic Syndrome

**DOI:** 10.7759/cureus.70159

**Published:** 2024-09-25

**Authors:** Shahzaib Fida, Sucheta Sharma

**Affiliations:** 1 Internal Medicine, Maidstone General Hospital, Maidstone, GBR; 2 Internal Medicine, Maidstone and Tunbridge Wells NHS (National Health Service) Trust, Maidstone, GBR

**Keywords:** atypical hemolytic uremia syndrome, eculizumab, hemorragic stroke, neuro-imaging, severe hypertension

## Abstract

Atypical haemolytic uraemic syndrome (aHUS) is a rare and complex condition characterized by systemic thrombotic microangiopathy resulting from complement dysregulation. While primarily affecting renal microvasculature, aHUS can present with multi-organ involvement, posing significant diagnostic and therapeutic challenges. We report the case of a 22-year-old female with a history of aHUS who developed a catastrophic haemorrhagic stroke. Her clinical course underscores the severe and unpredictable nature of aHUS, illustrating the critical need for heightened awareness of its potential neurological manifestations. aHUS is typically triggered by a combination of genetic predisposition and environmental factors such as infections or medications. This case highlights the necessity for comprehensive evaluation and prompt intervention in patients with aHUS presenting with atypical symptoms. The complexity of aHUS necessitates a multidisciplinary approach to diagnosis and management to mitigate morbidity and mortality.

## Introduction

Atypical haemolytic uraemic syndrome (aHUS) presents a complex clinical profile and a diagnostic conundrum in itself, for which essentially no globally accepted diagnostic criteria have been defined. This disease is characterized by the confluence of non-immune haemolytic anaemia, thrombocytopenia, and acute renal failure. In children, aHUS affects boys and girls equally. However, in adults, women are more likely to be affected, possibly due to pregnancy triggering the condition. The exact rate of aHUS is not well known. This syndrome revolves around systemic thrombotic microangiopathy (TMA), which manifests through a cascade of pathological events. Various etiological factors (genetics, pregnancy, cancer, and infections like certain strains of *Escherichia coli*) can trigger the TMA process that typifies HUS [1]. Specifically, within the spectrum of HUS subtypes, aHUS emerges as a distinct entity, wherein the TMA cascade is precipitated by endothelial injury within the microvasculature of vital organs, primarily the kidneys. This endothelial disruption is attributed to dysregulation within the complement system, a crucial component of the body's immune response. Recent advancements in genetic research have illuminated the landscape of aHUS, with a plethora of mutations identified in genes governing the complement system The central mechanism involves microangiopathy and endothelial injury, which are a result of heightened C5 activation and the formation of the membrane attack complex (MAC). This process leads to platelet activation, aggregation, and the release of microparticles that express tissue factors, contributing to the formation of thrombi in the microvasculature. The resulting endothelial damage causes thrombus formation, platelet consumption, and red blood cell fragmentation [2]. Other cases of aHUS are linked to loss-of-function mutations in genes encoding complement regulatory proteins such as membrane cofactor protein and factor I. Additionally, gain-of-function mutations in genes encoding key complement proteins like complement factor B and C3 are also associated with aHUS. Mutations in the gene for thrombomodulin (THBD), an endothelial anticoagulant glycoprotein with complement regulatory roles, have been identified in 3-5% of aHUS patients. aHUS arises from genetic alterations affecting the regulatory mechanisms of the immune system. However, the mere presence of a genetic mutation is typically insufficient to precipitate the onset of the disease. Rather, a confluence of factors, colloquially termed as "triggering events," is often required to instigate symptomatic manifestations of aHUS within the body's physiology. 

It is noteworthy that not all individuals diagnosed with aHUS exhibit detectable genetic mutations through conventional testing methodologies. This discrepancy is postulated to stem from the incomplete elucidation of all gene mutations associated with aHUS, suggesting a broader genetic landscape yet to be fully characterized by ongoing research endeavours. 

aHUS is a multifaceted disorder characterized by various clinical symptoms and complications. While the renal microvasculature is frequently affected by TMA, aHUS can also present in various organ systems and resemble multiple other conditions [3]. Due to the multi-organ involvement in aHUS, clinical manifestations can include cardiovascular, neurological, dermatological, abdominal, and peripheral vascular symptoms [3]. Due to the significant morbidity and mortality linked to aHUS, it is essential to understand the possible extrarenal manifestations and related laboratory findings to ensure timely diagnosis and treatment. In this case report, we present the case of a 22-year-old female with a history of aHUS, who presented with the catastrophic sequelae of the disease, specifically a haemorrhagic stroke.

## Case presentation

The patient is a 22-year-old female independent in her daily activities with a past medical history of the following: 

1. aHUS - On eculizumab every two weeks - under the care of Evelina hospital, diagnosed in 2010 (Factor H auto-antibody strongly positive, CF1 - normal levels but with a sequence variation in exon 3 on CSI)

2. Chronic kidney disease - stage 3 

3. Hypertension - managed with spironolactone, doxazosin, amlodipine, lisinopril, and atenolol. 

**Figure 1:**
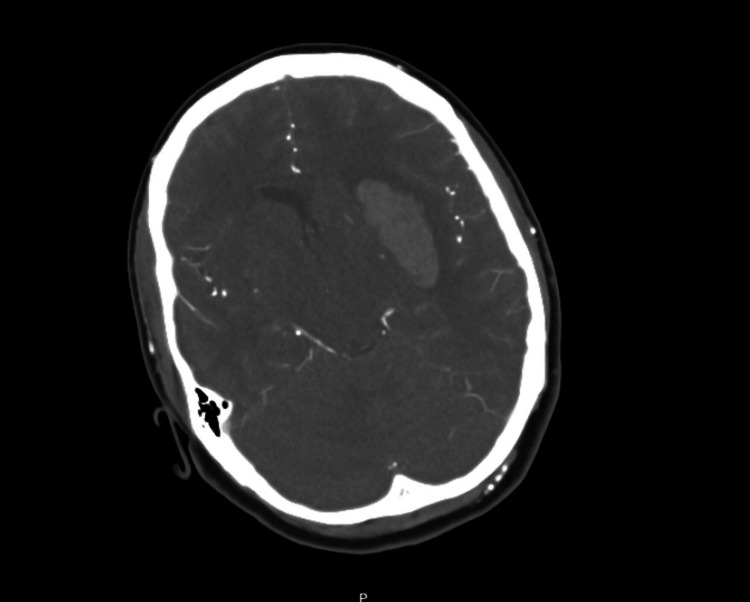
CT Head (non-contrast) showing intracerebral haematoma in the left internal capsule

She presented to the emergency department with right-sided weakness and facial droop. Upon arrival at the emergency department, the patient was noted to have a significantly elevated blood pressure of 244/166 mmHg. Further history revealed that she had received an Ozempic injection the previous day and had felt unwell since then, experiencing nausea and multiple episodes of vomiting throughout the night. The next morning, the patient's mother found her non-verbal with a right-sided facial droop and weakness in her right arm and leg. Her blood pressure at that time was recorded as greater than 250 mmHg by paramedics. After the patient was brought to A&E, she was initially assessed by the Stroke team. On initial assessment, the patient had a fluctuating Glasgow Coma Scale (GCS), was opening eyes a few times, and was almost non-verbal. There was no obviously evident facial droop but displayed severe expressive and receptive dysphasia. No movement was noted in the right upper and lower limbs, with a drift in the left arm, and the patient was able to move her left leg independently. Her NIHSS (National Institutes of Health Stroke Scale Score) was 28 at the time of presentation and she was immediately taken for CT Head (Figure 1). CT Head showed a large, acute, intracerebral haematoma centred on the left external capsule (47 x 34 x 19 mm).

**Figure 2:**
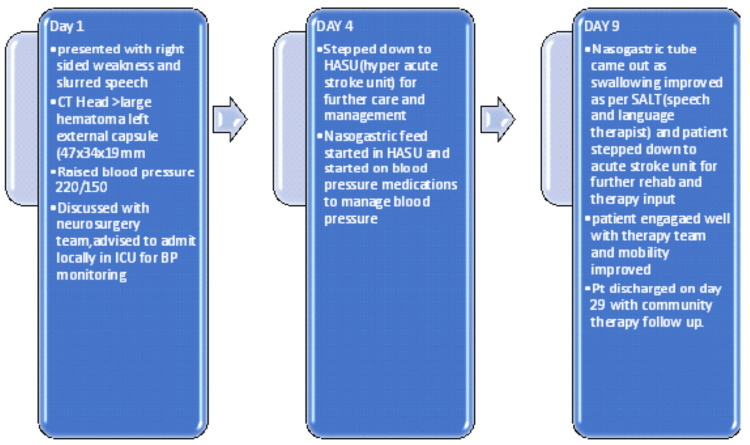
Timeline of clinical events

Accompanying vasogenic oedema and mass effect were noted as well. There was no evidence of blood within the ventricles or subarachnoid space. The images were discussed with the neurosurgical team at King's College Hospital as per the trust protocol for acute intracranial haemorrhage. Still, she was deemed unsuitable for surgical intervention and they advised to manage locally by the stroke team. However, the team at KCH agreed for her to be transferred in case of further deterioration. She was subsequently transferred to the intensive care unit (ICU) for blood pressure management. The patient's biochemical investigations at the time of presentation were unremarkable except for raised creatinine which was her baseline given chronic kidney disease (CKD) (Table 1). The coagulation screen was normal and non-indicative of any active ongoing coagulopathy. In the ICU, the blood pressure was controlled (with the aim of systolic blood pressure <140) with dual antihypertensive therapy, glyceryl trinitrate (GTN) and labetalol, in addition to her regular antihypertensive medications, which included spironolactone, doxazosin, amlodipine, lisinopril, and atenolol. Following the stabilization of her blood pressure, GTN and labetalol were stopped and she was continued on oral anti-hypertensives. The patient was transferred to the High Acuity Stroke Unit (HASU) for continued blood pressure management and therapy input. CT angiogram was performed a day later to look for any sites of potential bleeding, for example, intracranial aneurysms but it showed no signs of aneurysms but an unchanged appearance of the left intracerebral haematoma sparing the focal oedema sphincter resulting in the effacement of the left lateral ventricle with contralateral midline shift of 3.0 mm along with significant stenosis or dilatation of the intracranial vessels. The patient was initially started on nasogastric feed but as her swallowing improved insidiously over time, the NG tube was taken out within two weeks and the oral feed was introduced gradually. The chronology of the events is well depicted in Figure 2. The patient had regular reviews and inputs from the physiotherapy and occupational therapy teams. The patient engaged well with the therapy and showed a remarkable improvement in terms of her speech and neurological weakness gradually over 3-4 weeks. During hospitalization, she was investigated for the underlying cause of haemorrhage and secondary hypertension. The recommended tests done were as follows - urine meta-adrenaline, urinary nor-metadrenaline, and urine 3-methoxytyramine levels - which came back in the normal range (Table 2). USG Doppler of kidneys was requested which did not happen during her course of stay. Given the unremarkable blood findings, it was likely that her impaired blood pressure control was attributed to chronic renal damage caused by aHUS.

**Table 1:** Biochemical investigations CRP, C-reactive protein; ALT, alanine aminotransferase.

Biochemical investigation	Result	Normal range
CRP	4	<5 mg/L
Haemoglobin	132	120-150 g/L
White cell count	9.43	4.00-10.00 × 10^9^/L
Platelet count	267	150-410 × 10^9^/L
Sodium	140	133-146 mmol/L
Potassium	4.0	3.5-5.3 mmol/L
Creatinine	213	44-80 umol/L
Total bilirubin	4	<21 umol/L
Alkaline phosphatase	85	30-130 U/L
Serum albumin	43	35-50 g/L
ALT	14	<35 U/L

**Table 2:** Urine analysis results

Urine report on 1/5/24	Result	Normal value
pH urine	1.5	
Urine volume	0.854	-
Urine normetadrenaline	0.49	3.70 umol/24 h
Urine metadrenaline	0.5	<1.30 umol/24 h
Urine 3-methoxytyramine	0.33	<2.60 umol/24 h

The patient was discharged before the ultrasound Doppler and the GP was asked to follow up on ultrasound. She continued on eculizumab fortnightly as per her usual aHUS treatment. Her blood pressure medications were held during admission due to consistently low blood pressure. The patient improved and was discharged from the hospital with community physiotherapy and speech team input at home. She was followed up two months post-presentation in the stroke clinic and no new concerns were identified; hence, the patient was discharged from the stroke team.

## Discussion

**Figure 3:**
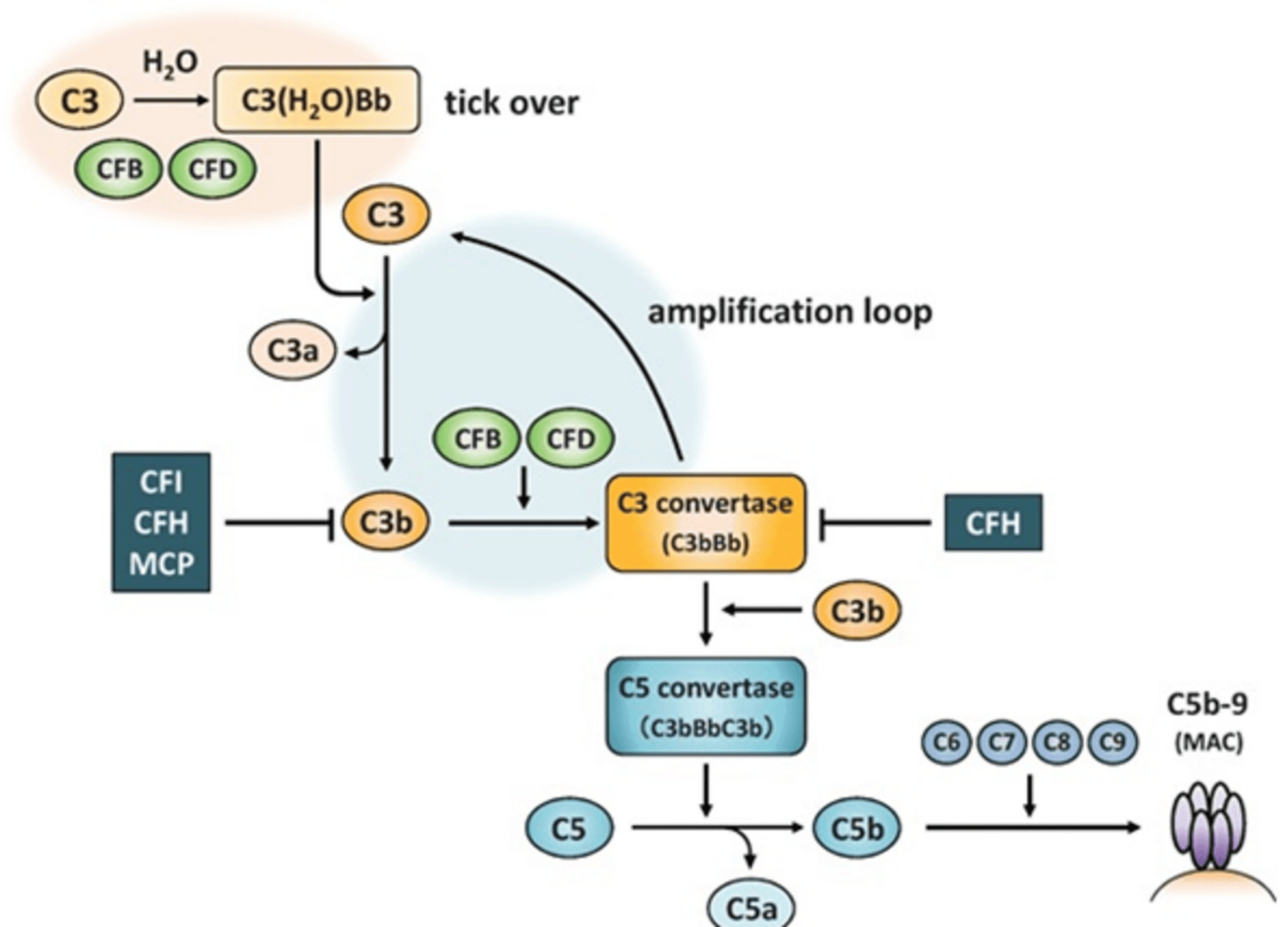
Alternative pathway of CFB, CFD, CFI, CFH, MCP, MAC (genes associated with aHUS) CFB: complement factor B; CFD: complement factor D; CFI: complement factor I; CFH: complement factor H; MCP: membrane cofactor protein; MAC: membrane attack complex; aHUS: atypical haemolytic uraemic syndrome.

TMAs often seen in clinical practice include thrombotic thrombocytopenic purpura (TTP) and haemolytic uremic syndrome caused by Shiga toxin-producing *E. coli* (STEC-HUS). Less common TMAs are aHUS and secondary HUS [4]. A key difference between aHUS and STEC-HUS is that aHUS is linked to mutations in the complement factor H (CFH) gene, which helps regulate the complement system in the blood [5]. Even though complement system problems are important in aHUS, normal levels of complement components C3 and/or C4 don't rule it out, so these biomarkers are not reliable for diagnosis [2]. Our patient had a positive Factor H auto-antibody and normal complement factor I levels but a variation in exon 3 of CSI (Figure 3).

To make diagnosing aHUS easier and improve patient care, the Japanese Society of Nephrology and the Japan Paediatrics Society (JSN/JPS) created criteria based on three main features: low platelet count (below 150,000/μL), microangiopathic haemolytic anaemia (shown by low haemoglobin, high lactate dehydrogenase, low haptoglobin, and fragmented red blood cells in a blood smear), and acute kidney failure (as per Kidney Disease: Improving Global Outcomes guidelines) [6]. Our patient had kidney failure and was diagnosed with aHUS through extensive genetic testing, even though she didn't have anaemia or low platelet count and had no family history of the illness. 

The symptoms of aHUS depend on the affected organ. Hypertension is common, resulting from decreased kidney blood flow due to microthrombi. Cardiovascular complications may include cardiomyopathy or myocardial infarction, while neurological complications can manifest as headaches, double vision, irritability, drowsiness, facial paralysis, seizures, transient ischemic attacks, stroke, and coma. Gastrointestinal bleeding and pulmonary complications like bleeding or pulmonary oedema are also possible. Blood pressure was over 250 mmHg, and a haemorrhagic stroke was diagnosed, an uncommon presentation in aHUS. aHUS primarily affects the kidneys, but extra-renal complications are also common. Up to 25% of patients may have persistent complications like hypertension and chronic renal failure, even decades after apparent recovery. About a third of cases involve ongoing kidney damage, making long-term monitoring essential. The most accurate predictor of long-term prognosis is a renal biopsy [7], and the findings commonly elicited on renal biopsy are 50% of destroyed glomeruli by microangiopathic damage, arterial microangiopathy, and cortical necrosis. 

Clinical manifestations and complications 

Neurologic complications are the most frequent non-renal manifestation in aHUS, observed in 8-48% of cases [8-12]. Common symptoms include seizures, vision loss, hemiparesis, headaches, altered consciousness, hallucinations, and encephalopathy. Other reported neurologic features are confusion, agitation, decreased reflexes, nystagmus, double vision, hemiplegia, focal neurologic deficits, and coma [2,8,9,12,13]. Cerebral imaging in TMA can reveal various changes, including changes in the posterior white matter, cortex, deep white matter, thalami, brainstem, and basal ganglia [14,15]. Neurologic involvement presents a wide range of manifestations, making it difficult to distinguish aHUS from other TMAs like TTP, which is caused by a deficiency in ADAMTS13 [16] activity. Proper management of factors like uraemia, electrolyte imbalances, hypertension, ischaemia, and cerebral oedema can reduce the risk of long-term neurologic complications.

Cardiovascular complications in aHUS have been reported in 3-10% of cases [9,17]. A review of 169 paediatric aHUS patients in Turkey [18] noticed cardiac features in 7% of cases, including left ventricular hypertrophy, cardiomyopathy, valve insufficiency, and tachycardia. Cardiac dysfunction may be linked to hypertension and fluid overload from acute kidney injury or may occur independently, indicating direct myocardial and vascular injury [19]. Microangiopathic injury in the coronary vessels can cause the thickening of small vessel walls and subendothelial swelling, increasing the risk of severe cardiac dysfunction and sudden cardiac death [20].

Dermatologic and peripheral vascular manifestations are rare in aHUS but can include severe skin changes and peripheral ischaemia, often responding quickly to plasma exchange and/or eculizumab [21-24]. Pulmonary complications in aHUS usually happen along with multiple organ dysfunction and pulmonary congestion due to heart problems or too much fluid in the body [25]. Other lung issues like pulmonary haemorrhage [26] and pulmonary embolism [27] have also been reported in a few cases. In a review by Johnson et al. [11] of 71 children with aHUS, 15 patients (21%) developed severe breathing problems needing mechanical ventilation. While direct lung damage is rare, respiratory failure due to lung swelling is a serious and dangerous issue in aHUS. Therefore, it is important to closely monitor fluid and breathing to prevent lung problems in these patients.

Management and outcomes

Since aHUS is a multi-organ disorder with potentially fatal complications, continuous monitoring and follow-up are crucial. Even when hospitalized, these patients should receive prioritized, close observation. In terms of pharmacological management, eculizumab is a highly effective treatment that blocks part of the complement system, stopping the disease process [28]. It is recommended as the first treatment for children due to their higher risk of complications with TPE. Guleroglu et al. reported two paediatric aHUS cases with notable neurological symptoms and abnormal MRI findings [14]. The first was an 11-year-old girl who had multiple seizures and MRI changes in the occipital and posterior parietal lobes a month after diagnosis. She recovered completely with eculizumab treatment. The second case involved a six-year-old girl who, after initial symptoms of vomiting and abdominal pain, developed seizures, nystagmus, and vision loss. Her MRI showed hyperintense lesions in the same brain regions, and she also recovered completely with eculizumab and had no residual deficits. Neurological symptoms often improve quickly after starting eculizumab [12,14,29]. Tsai et al. noted that four out of five adults with altered mental status saw resolution within seven days of treatment [30]. Additionally, an eight-year-old boy with anti-Factor H antibody-associated aHUS fully recovered, with an MRI showing brain lesion regression three months later [12]. The patient discussed in this case report had plasma exchange for one year as eculizumab was not licensed to use in the UK at that time [31] and afterwards, she was started on eculizumab in 2013 and since has been on eculizumab fortnightly without a miss until she was presented to A&E. The long-term outcome as discussed earlier is predicted based on the renal biopsy results of the patient. 

## Conclusions

aHUS causing a haemorrhagic stroke is a very rare diagnosis. The prognosis and long-term complications of individuals with aHUS can be significantly improved by early recognition and comprehensive management. A multidisciplinary approach is essential for aHUS due to its rarity and potential severity. Ongoing research into the genetic and pathophysiological underpinnings of aHUS is essential to improve diagnostic accuracy and treatment outcomes.
